# Automatic Foreground Extraction Based on Difference of Gaussian

**DOI:** 10.1155/2014/296074

**Published:** 2014-07-20

**Authors:** Yubo Yuan, Yun Liu, Guanghui Dai, Jing Zhang, Zhihua Chen

**Affiliations:** ^1^Department of Computer Science and Engineering, East China University of Science and Technology, Shanghai 200237, China; ^2^State Key Lab for Novel Software Technology, Nanjing University, Nanjing 210093, China

## Abstract

A novel algorithm for automatic foreground extraction based on difference of Gaussian (DoG) is presented. In our algorithm, DoG is employed to find the candidate keypoints of an input image in different color layers. Then, a keypoints filter algorithm is proposed to get the keypoints by removing the pseudo-keypoints and rebuilding the important keypoints. Finally, Normalized cut (Ncut) is used to segment an image into several regions and locate the foreground with the number of keypoints in each region. Experiments on the given image data set demonstrate the effectiveness of our algorithm.

## 1. Introduction

In the image processing, the foreground is an integral part of the objective image. It takes important advantage in many applications [1–4]. For example, in the field of object recognition, in 2011, Rosenfeld and Weinshall [5] proposed an algorithm to extract a foreground mask and to identify the locations of objects in the image. In the field of object tracking, in 2012, Wang et al. [6] used partial least squares (PLS) analysis to label the foreground and background of an image and the results showed that the proposed tracking algorithm was very powerful with the labeled foreground. In the field of content-based image retrieval, in 2006, Shekhar and Chaudhuri [7] investigated the influence of the foreground. In the field of image editing, in 2008, Levin et al. [8] indicated that the process of extracting a foreground object from an image based on limited user input was an important task in image editing.

In current methods, foreground extraction can be classified into two categories [9], one is the interactive foreground extraction and the other is the automatic one. The interactive foreground extraction can accurately find artificial areas from the input images; however, it can do nothing when the task is to extract foregrounds from thousands of images. In this case, the technology of extracting foregrounds automatically is becoming more and more important. Moreover, it can be applied in many fields, such as image segmentation, image enhancement, object recognition, and content-based image retrieval.

In 2011, Kim et al. [10] proposed an automatic method to extract a foreground object captured from multiple viewpoints. Their result was the high quality alpha mattes of the foreground object consistently across all different viewpoints. In 2012, Zhang et al. [11] proposed a technique of automatic foreground-background segmentation based on depth from coded aperture. Their entire progress was fully automatic, without any manual intervention. In 2013, Hsieh and Lee [12] proposed an automatic trimap generation technique. The experimental results showed that the trimap generated by the proposed method effectively improves the matting result. Moreover, they processed the enhancement of the accuracy of the trimap results in a reduction of regions, so that the extraction procedure can be accelerated.

In recent years, with the development of video technology, the research on the automatic foreground extraction will become more and more popular. In this paper, we present a novel approach for automatic foreground extraction based on difference of Gaussian. We employ the difference of Gaussian (DoG) to find candidate keypoints. After filtering and rebuilding the candidate keypoints, we get the refined keypoints of an input image. With Ncut, we extract the foreground from the original image.

The rest of this paper is organized as follows. In Section 2, we introduce the key steps of proposed extraction algorithm, including basic framework, candidate keypoints locating, and keypoints filter. In Section 3, the algorithm is presented. In Section 4, some excellent experimental results are shown. Finally, we give a conclusion with this research work.

## 2. Foreground Extraction Based on Difference of Gaussian

### 2.1. Framework

The motivation of this work is to develop a useful technology to extract the foreground from an input image. The contributions of this paper are as follows.

- A new procedure to find candidate keypoints in different color layers is proposed. It helps us to find the point that has a more obvious color difference than its neighbours. It is implemented efficiently by using a difference of Gaussian function to find candidate keypoints. We fulfill this task in different color layers.
- Novel filtering operators are constructed to remove the pseudo-keypoint and rebuild the important keypoints. This stage can be summarized into two steps. The first is to reduce the candidate keypoints to find the edge of the foreground by the result of Ncut. Another step is to rebuild the points to increase the proportion of candidate keypoints by a novel approach. At last, we call these candidate points keypoints.
- Novel operator for foreground extraction is proposed. We locate the foreground by the proportion of keypoints and segment the foreground by the result of Ncut.

**Figure 1:**
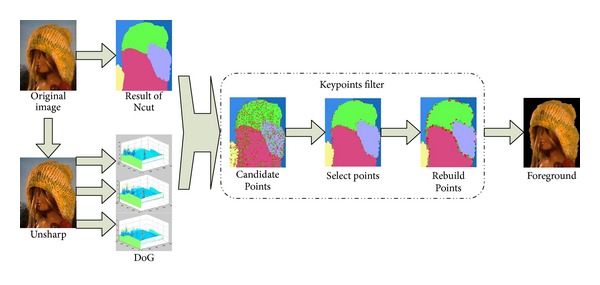
Flowchart of the basic procedure of FMDOG. The result of difference of Gaussian (DoG) and Normalized cut (Ncut) is combined by the keypoints filter. The last image is the foreground we extract in this model image.

The basic procedures to locate the foreground are illustrated as in Figure 1.

### 2.2. Regional Segmentations Based on Normalized Cut

In this work, we use a well-known technique to segment the input image, namely, the Ncut method [15]. Based on the studies in [15], Ncut is from graph-partitioning method. We map the image into a graph **G** = (**V**, **E**, **W**), where **G**  =  (**V**) is the set of nodes, and **G**  =  (**E**) is the set of edges connecting the nodes. A pair of nodes *u* and *v* is connected by edge and is weighted by *w*(*u*, *v*) = *w*(*v*, *u*) ≥ 0 to measure the dissimilarity between them. The basic optimal model of graph cut can be given by

(1) $$\begin{array}{c} C^{*}=\arg\underset{C\subset V}{\min}\left\{\text{cut}\left(C\right)\right\}, \end{array}$$

in which

(2) $$\begin{array}{c} \text{cut}\left(C\right)=\underset{u\in C,v\in V-C}{\sum }w\left(u,v\right). \end{array}$$

In [15], Shi and Malik proposed Ncut method based on the following optimal model:

(3) $$\begin{array}{c} C^{*}=\arg\underset{C\subset V}{\min}\left\{\text{Ncut}\left(C\right)\right\}, \end{array}$$

in which

(4) $$\begin{array}{c} \text{Ncut}\left(C\right)=\frac{1}{\text{normal}\left(C\right)}\text{cut}\left(C\right), \end{array}$$

where

(5) $$\begin{array}{c} \text{normal}\left(C\right)=\frac{\text{assoc}\left(C,V\right)\cdot \text{assoc}\left(V-C,V\right)}{\text{assoc}\left(C,V\right)+\text{assoc}\left(V-C,V\right)}, \end{array}$$

in which assoc(**C**, **V**) denotes the total connection from nodes in **C** to all nodes in the graph and assoc(**V** − **C**, **V**) is similarly defined.

In practice, Ncut is a powerful segmentation method. After employing the Ncut algorithm, if we denote the *j*th part of the *i*th input image as **R**_*ij*_, the input image **I**_*i*_ is segmented into *M*_*i*_ parts

(6) $$\begin{array}{c} I_{i}=\overset{M_{i}}{\underset{j=1}{⋃}}R_{ij}. \end{array}$$

### 2.3. Candidate Keypoints Locating

An input image *I*(*x*, *y*) can be seen as a surface on square domain Ω = [*a*, *b*]×[*c*, *d*]. In the discrete space, if the total number of pixels is *m* × *n*, the domain can be represented as {1,2,…, *m*}×{1,2,…, *n*}.

Candidate keypoints of an input image are detected firstly by difference of Gaussian (DoG) algorithm. The difference of Gaussian with the scale *σ* and constant multiplicative factor *k* can be computed by

(7) $$\begin{array}{c} D\left(x,y,\sigma ,k\right)=L\left(x,y,k\sigma \right)-L\left(x,y,\sigma \right),\;\left(x,y\right)\in \frac{\Omega }{\partial \Omega }, \end{array}$$

in which *L*(*x*, *y*, *σ*) is the scale space and ∂Ω denotes the boundary of the input image. *L*(*x*, *y*, *σ*) can be obtained by

(8) $$\begin{array}{c} L\left(x,y,\sigma \right)=G\left(x,y,\sigma \right)*I\left(x,y\right), \end{array}$$

where ∗ is the convolution operation and

(9) $$\begin{array}{c} G\left(x,y,\sigma \right)=\frac{1}{2\pi \sigma^{2}}e^{-(x^{2}+y^{2})/2\sigma^{2}}. \end{array}$$

In practice, the size is usually chosen as *σ* = 1.6 and the constant multiplicative factor is chosen as $k=\sqrt{2}$.

We will detect maxima and minima of the difference of Gaussian images by comparing a pixel to its 26 neighbors in 3 × 3 regions at the current and adjacent scales. Once the value of a pixel is maxima or minima, we regarded this pixel as a* candidate point*. Mathematically, for any (*x*, *y*) ∈ Ω, we get the map (*g*(*x*, *y*)) of the candidate points, where

(10) g(x,y)={1,the  value  of  g(x,y)is  maxima  or  minima;0,otherwise.

### 2.4. Keypoints Filtering

In general, the candidate points can not be used to detect right foreground regions. They always gather in the areas with mixed colors. However, these areas may be in the background. Motivated by this observation, a point filtering function is constructed to reduce the candidate points in the background and rebuild some new candidate points in the foreground. We call these candidate points as keypoints.

The filtering function is formulated as

(11) $$\begin{array}{c} f\left(x,y\right)=\{\begin{array}{cc} 0, & S\left(x,y\right)>1;\\ 1, & S\left(x,y\right)\leq 1, \end{array} \end{array}$$

in which *f*(*x*, *y*) = 1 means that (*x*, *y*) is the candidate point; otherwise (*x*, *y*) is not. The selection function can be formulated as follows:

(12) $$\begin{array}{c} S\left(x,y\right)=\underset{x-2\leq i\leq x+2,y-2\leq j\leq y+2}{\sum }g\left(i,j\right). \end{array}$$

**Remark 1 .**

We create a 5 × 5 filter to reduce the candidate points. Only one candidate point can remain in the 5 × 5 filter.

Through the filter, the number of the candidate points is decreased dramatically. In particular, for the dense candidate points in one region, the reduction is obvious.

In the next step, we employ the Ncut to find the edges of an input image. With Ncut, we segment the image into many regions and locate the edge points.

We select the more useful candidate points along the edges. We keep the candidate points on the edges. This process can be formulated as follows:

(13) $$\begin{array}{c} c\left(x,y\right)=\{\begin{array}{cc} 1, & f\left(x,y\right)=1,\left(x,y\right)\;\text{is}\;\;\text{one}\;\;\text{edge}\;\;\text{point};\\ 0, & \text{otherwise}. \end{array} \end{array}$$

In the next step, we will rebuild some new candidate points.

Firstly, we need to define the focus of an image. We regard center of the region that contains the largest number of candidate points as the focus. The region that the focus in (*R*_*f*_) can be computed by

(14) $$\begin{array}{c} R_{f}=\{\begin{array}{cc} R_{i}, & \max\left(\underset{\left(x,y\right)\in R_{i}}{\sum }c\left(x,y\right)\right)>\underset{\left(x,y\right)\notin R_{i}}{\sum }c\left(x,y\right);\\ R, & \text{otherwise}, \end{array} \end{array}$$

where *R*_*i*_ is the *i*th region of an input image and

(15) $$\begin{array}{c} R=⋃R_{i}. \end{array}$$

The initial focus is located in the center of the image. The focus may shift to the other regions that contains the largest number of candidate points.

The candidate points will be rebuilt towards the focus. For an input image, there are four boundaries. We reserved the boundary information included in each area in the candidate points of this area. Some weight is added to each already existed point. The weight is determined by the boundary information of the candidate point. The more boundaries it contains, the smaller the weight is. The weight can be computed by

(16) w(x,y)={1,the  region  this  point  in  contains 0 boundary;0.5,the  region  this  point  in  contains 1 boundary;0,otherwise.

**Figure 2:**
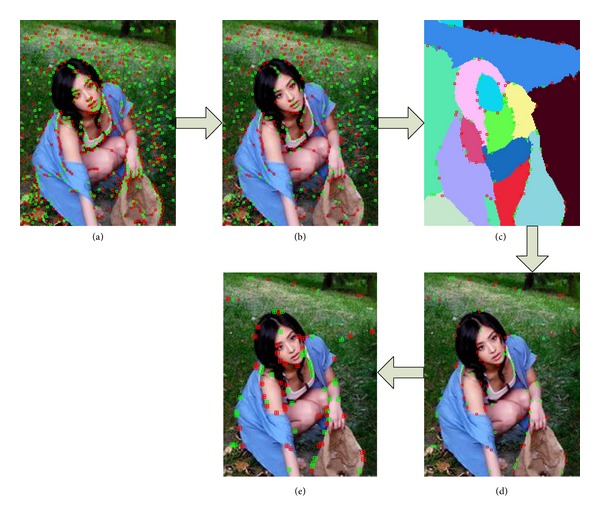
Basic procedure of the keypoints filter. (a) Initial result of difference of Gaussian (DoG). (b) Candidate points we get through the 5 × 5 filter. (c) Information of edges. (d) Information of edges on the original image. (e) Result of candidate points rebuild.

We regard the candidate points which are obtained from the above steps as keypoints. At last, we locate the foreground by the number of keypoints in each region. The process of filtering keypoints is displayed in Figure 2.

## 3. Proposed Algorithm to Extract the Foreground

In this section, we give the basic procedures of our proposed algorithm. The main steps include difference of Gaussian (DoG), generation of candidate points, keypoints filtering, and locating the foreground. The pseudo-codes are listed in Algorithm 1.

**Remark 2 .**

In Algorithm 1, *thresh* and *NcutNum* are two constants given by users or experts. Otherwise, they can be determined by a learning procedure. In this paper, *thresh* = 0.2 and *NcutNum* = 12 are determined by the observation value on many experimental results.

**Remark 3 .**

It is a big trouble problem for noisy images, because it is very difficult to detect the keypoints with DoG. In this case, we need to employ the operator to move the noise away at the beginning step of this algorithm.

## 4. Experiments

### 4.1. Images Data Set

We evaluate our extraction technique in two different data sets. The first data set consists of 27 images that are the most popular images used to extract foreground interactively. The second one is created by ourselves which contains 26 images. The latter one is more complicated than the front.

**Figure 3:**
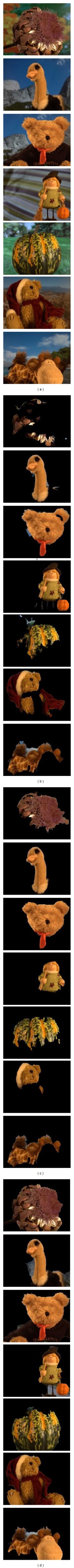
Results in the first data set. (a) Original images. (b) Foreground extracted with RC [13]. (c) Foreground extracted with TSS [14]. (d) Foreground extracted with FMDOG.

**Figure 4:**
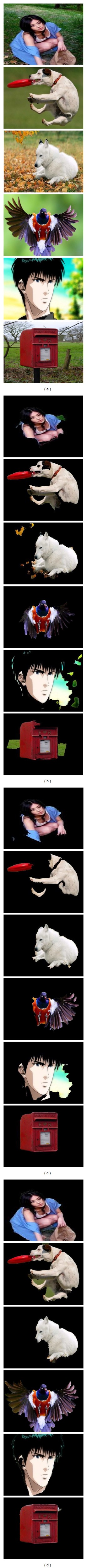
Results in the second data set. (a) Original images. (b) Foreground extracted with RC [13]. (c) Foreground extracted with TSS [14]. (d) Foreground extracted with FMDOG.

Some excellent results in the first data set are shown in Figure 3 and the other excellent results in the second data set are shown in Figure 4. Observations on Figures 3 and 4, our proposed algorithm, can extract the foreground beyond 95%. The original images in Figure 3 have rich color and texture in the foreground. When we use the DoG to check out the candidate keypoints, the number of keypoints in the foreground is larger than in the background. In this case, it is easy to extract the foreground. However, few of the images in the first data set are difficult to extract foreground automatically because there is confusion between foreground and background. For the images with outstanding target and complicated background, although more keypoints in the background are obtained, we can get effective keypoints by the keypoints filtered function. So we can extract the foreground with high performance, for example, the lady, the dog, and the postbox.

In order to the effectiveness of our proposed method, two good algorithms are employed to extract the foreground from our 27 images. They are well-known in the saliency detection. One is regional contrast (RC) method and the other is two-stage scheme (TSS) method.

### 4.2. Regional Contrast Based Saliency Extraction Algorithm (RC)

Cheng et al. [13] proposed a regional contrast based saliency extraction algorithm (RC), which simultaneously evaluates global contrast differences and spatial coherence. Their algorithm was simple, efficient and yields full resolution saliency maps.

At the beginning of the algorithm, an input image is mapped into a graphic, which is used to segment the image by GB [16]. The mapping operator is as follows:

(17) I⟶G=(V,E,W),

where **V** denotes the pixel in the image. **E** denotes the edge connected between adjacent pixels. **W** is the weight of the edge.

With the minimum spanning tree and the smallest weight value between two vertexes, the input image is segmented into several regions as follows:

(18) I={s0,s1,…,sK},

in which the number *K* means that the image is mapped into *K* parts.

For each segmented region, its salient values are calculated by comparing itself with the value of the other regions in Lab color space. In the same region, each pixel has the same salient value. The spatial distance information is also the important factor that influences salient value, so we consider it in the saliency detection. If one segmented region is close to current segmented region, the saliency influence of it is big. Otherwise, the influence is small.

The formula that adds spatial weights is as follows [13]:

(19) $$\begin{array}{c} \bar{S}\left(s_{k}\right)=\underset{s_{k}\neq s_{i}}{\sum }\exp\left(-\frac{D_{s}\left(s_{k},s_{i}\right)}{\sigma_{s}^{2}}\right)w\left(s_{i}\right)\bar{D_{s}}\left(s_{k},s_{i}\right), \end{array}$$

where *w*(*s*_*i*_) is the weight value of region *s*_*i*_, which is defined as the number of pixels in *s*_*i*_. *D*_*s*_(*s*_*k*_, *s*_*i*_) is the distance between region *s*_*k*_ and *s*_*i*_, which is defined as the Euclidean distance between their centers of gravity. *σ* ^2^ is used to control the strength of spatial weight. If the value *σ* ^2^ is big, the impact of the spatial weight is great, and the region far from the current region will have a stronger impact. Here, *σ* ^2^ is set as 0.4, and the pixel coordinates are all normalized to [0,1]. ${\bar{D}}_{s}(s_{k},s_{i})$ is the color distance metric between *s*_*k*_ and *s*_*i*_.

The color distance formula between *s*_1_ and *s*_2_ is defined as follows [13]:

(20) $$\begin{array}{c} {\bar{D}}_{s}\left(s_{1},s_{2}\right)=\overset{n_{1}}{\underset{i=1}{\sum }}\overset{n_{2}}{\underset{j=1}{\sum }}f\left(s_{1,i}\right)f\left(s_{2,j}\right)D\left(s_{1,i},s_{2,j}\right), \end{array}$$

in which *f*(*s*_*k*,*i*_) is the frequency of *i*th color *s*_*k*,*i*_ among all *n*_*k*_ colors in *k*th segmented region *s*_*k*_, *k* = {1,2}.

### 4.3. Two-Stage Scheme for Bottom-Up Saliency Detection (TSS)

In 2013, Yang et al. [14] proposed a two-stage scheme (TSS) for bottom-up saliency detection using ranking with background and foreground queries. In this subsection, we introduce the basis procedures and the primal ideas of the TSS. The following sentences are referred from [14].

At first, an input image is represented as a close-loop graph with super-pixels as nodes (a graph **G**  =  (**V**, **E**, **W**) with super-pixels as nodes, **V** is a set of nodes and **E** is a set of undirected edges). The weight between two nodes is defined by

(21) wij=e−||ci−cj||/2, i,j∈V,

in which *c*_*i*_ and *c*_*j*_ denote the mean of the super-pixels corresponding to two nodes in the feature space and is a constant that controls the strength of the weight.

The graph-based ranking technique is employed to calculate the similarity of the image elements (pixels or regions) with foreground cues or background cues. The basic idea is that for a given node as a query, the remaining nodes are ranked based on their relevances to the given query. The goal is to learn a ranking function, which defines the relevance between unlabelled nodes and queries. The saliency of the image elements is defined based on their relevances to the given seeds or queries. The saliency map of the first stage is binary segmented (i.e., salient foreground and background) using an adaptive threshold, which facilitates selecting the nodes of the foreground salient objects as queries. The selected queries cover the salient object regions as much as possible (i.e., with high recall). The threshold is set as the mean saliency over the entire saliency map. Once the salient queries are given, an indicator vector **y** is formed to compute the ranking vector **f** using the equation

(22) $$\begin{array}{c} f=Ay. \end{array}$$

In (22), **A** can be regarded as a learnt optimal affinity matrix and can be determined by the supervised manifold learning (details can be seen in Section 2.2 from [14]). As is carried out in the first stage, the ranking vector **f** is normalized between the range of 0 and 1 to form the final saliency map. It is calculated by

(23) $$\begin{array}{c} S_{fq}\left(i\right)=\bar{f}\left(i\right),\;i=1,2,\ldots ,N, \end{array}$$

where *i* indexes super-pixel node on graph and $\bar{f}$ denotes the normalized vector.

### 4.4. Results

The results of these methods are displayed in Figures 3 and 4. It is obvious that better results can be obtained by our method in most cases.

## 5. Conclusion

In this paper, a novel approach for automatic foreground extraction is proposed. It is based on the difference of Gaussian (DoG). We create a keypoints filter to obtain the keypoints which are used to locate the foreground region in the image. Normalized cut (Ncut) is used to cut the image into different regions and find the information of the boundaries. This approach can be better applied to the image of which foreground is easy to identify by interactive foreground extraction. So our experiments are taken on the data set for interactive foreground extraction.

## Figures and Tables

**Algorithm 1:**
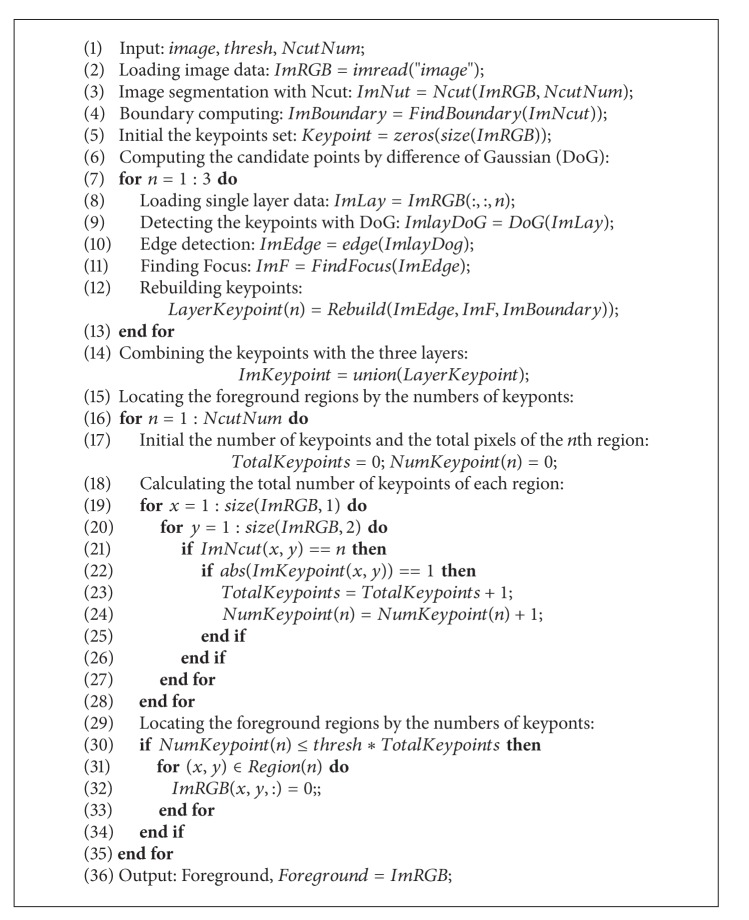
Extraction Algorithm.
